# Structural Analysis of Melt-Spun Polymer-Optical Poly(Methyl Methacrylate) Fibres by Small-Angle X-ray Scattering and Monte-Carlo Simulation

**DOI:** 10.3390/polym13050779

**Published:** 2021-03-03

**Authors:** Jan Kallweit, Thomas Vad, Felix Krooß, Thomas Gries, Mohmmed Houri, Christian-Alexander Bunge

**Affiliations:** 1 Institut für Textiltechnik, RWTH Aachen University, Otto-Blumenthal-Straße 1, 52074 Aachen, Germany; thomas.vad@ita.rwth-aachen.de (T.V.); felix.krooss@ita.rwth-aachen.de (F.K.); thomas.gries@ita.rwth-aachen.de (T.G.); 2 Hochschule für Technik, Wirtschaft und Kultur Leipzig (HTWK), Zschochersche Str. 69, 04229 Leipzig, Germany; mohmmed.houri1776@gmail.com (M.H.); christian-alexander.bunge@htwk-leipzig.de (C.-A.B.)

**Keywords:** material characterisation, polymer optical fibre, graded-index profile, fibre fabrication, nanostructure, measurement technique, scattering, melt-spinning

## Abstract

The structural properties, mainly the spatial variation of density and chain interaction, of melt-spun polymer optical fibres (POFs) are investigated by small-angle X-ray scattering (SAXS) and compared to Monte-Carlo polymer simulations. The amorphous PMMA POFs had been subjected to a rapid cooling in a water quench right after extrusion in order to obtain a radial refractive-index profile. Four fibre samples with different processing parameters are investigated and the SAXS data analysed via Guinier approach. Distance-distribution functions from the respective equatorial and meridional SAXS data are computed to extract the fibres’ nanostructures in the equatorial plane and along the fibre axis, respectively. Temperature profiles of the cooling process are simulated for different locations within the fibre and taken as input for Monte-Carlo simulations of the polymer structure. The simulation results agree with the SAXS measurements in terms of the cooling profile’s strong influence on the structural properties of the fibre: slower cooling in the centre of the fibre leads to stronger interchain interaction, but also results in a higher density and more homogenous materials with less optical scattering.

## 1. Introduction

Most large-core polymer optical fibres (POF) are made from poly-methyl methacrylate (PMMA). While they can provide a robust and cost-effective alternative to glass fibres for short-reach data communication, they are even better suited for illumination and sensing applications [1]. Depending on the kind of application, POFs should show different properties, such as controlled scattering for illumination and sensing or relatively low attenuation and small mode dispersion for data communication. Especially for the latter, graded-index (GI) fibres have been developed with a decreasing refractive-index profile towards the outer regions of the fibre [2,3,4]. Such a nonhomogeneous profile requires special fabrication methods, often involving a discontinuous preform processes [5].

We presented a novel, continuous fabrication process in [6], where the GI-POF is produced in a standard melt-spinning process with a water bath quench of the filament right behind the spinning nozzle instead of air cooling. No dopants are needed to control the refractive index. Different cooling rates of inner and outer regions result in a radial density gradient, which finally leads to a refractive-index profile. The approach to simplify GI-POF manufacturing offers the possibility to enhance the economic use of GI-POF and thus can be applied in many more fields to increase data transmission rates.

SAXS measurements have previously been carried out on PMMA-based fibres. In these cases, it was used for porosity measurement in mesoporous nanofibers [7], the analysis of additives in PMMA fibre nanocomposites [8] or a structural analysis of block copolymers with PMMA being one of the blocks [9]. SAXS experiments on pure PMMA POFs have not yet been performed mainly because amorphous materials like PMMA normally do not exhibit any small-angle scattering. Whereas the formation of a distinct refractive-index profile without any doping materials could be proven in [10], there are some open points concerning the quality of the fibre material and its potential attenuation due to scattering. A first small-angle X-ray scattering analysis of the structural properties of PMMA with rapid cooling was presented in [11]. SAXS measurements are ideally suited to detect nanoscale density fluctuations [12]. Therefore, SAXS data can be used to indirectly gain information about the origin of the obtained refractive-index profiles. The analysis concentrated on larger structures in order to find evidence for a refractive-index profile. Some ring-like structures were observed that can result in a decreasing refractive index towards the outer regions. In order to gain information on potential sources for optical scattering and other structure-related effects, the molecular structure has to be investigated on a smaller scale. Therefore, we analysed the SAXS measurements that had been presented in [11] once more and concentrated on different properties such as entanglement or interchain interaction and the variation of the density, which can both lead to scattering.

The article is structured as follows: firstly, the materials and the fabrication method are briefly described. Then, a simulation of the cooling process is presented, and the temperature profiles are extracted for the fibres under test at different locations along their radius. Afterwards, the experimental conditions and the data-analysis techniques for the SAXS measurements are expounded. Their results are discussed, and the basic assumptions for their analysis are adapted to explain the structural parameters of the different analyses. The findings indicate a higher chain interaction in the inner, more slowly cooled parts of the fibre. For a better understanding, a Monte-Carlo simulation of the cooling of PMMA was performed for the different temperature profiles and the resulting structural properties analysed. The numerical results also show a higher interaction between neighbouring polymers in the inner regions. The physical density is also higher and more homogenous. In summary, the temperature profiles during the cooling of PMMA show a strong influence on structural properties such as cross-chain interaction and physical density. SAXS is a suitable method for its analysis.

## 2. Adjusted Melt-Spinning Fabrication Process

Optical fibres, and POF in particular, can be fabricated in continuous or discontinuous processes. Continuous processes are preferred due to their low cost, but can only be applied to fibres with simple profiles such as step-index fibres. One well-known continuous method is melt spinning: the optical polymer is melted in the extruder and spun through a spinneret to form the fibre, which is cooled by air, wound up and eventually subsequently stretched. For fibres with more complex graded-index profiles, melt spinning is not suitable. For such a radius-dependent variation, usually a preform is needed, which is drawn to a fibre in a discontinuous second step [5].

### 2.1. Fundamental Principle

In order to achieve a graded-index profile in a continuous process, an adjusted melt-spinning technique can be used, where the cooling of the produced filament is dramatically faster in order to obtain different cooling speeds within the fibre. The process is described in detail in [13,14]. The different cooling speeds lead to different lengths of time for the polymer chains to align themselves energetically before the temperature drops below the glass-transition temperature. This results in a density gradient with an increase of the density for lower cooling speed in the centre of the fibre. According to the Clausius-Mosotti equation the density gradient can be translated into a similar refractive-index profile as shown in [15]. However, other properties of the polymer can also be influenced by the radius-dependent cooling, both on a molecular and macroscopic level. Therefore, polymer properties are investigated experimentally and numerically in the following.

### 2.2. Fibre Fabrication

The fibres for the SAXS measurements were the same filament samples as in [11]. These were examined again for much smaller structural properties than before in order to gain insight on the polymer alignment, chain interaction and structure on the molecular level. They had been produced with the PMMA grade PLEXIGLAS^®^ POQ66 (Röhm GmbH, Darmstadt, Germany). All four samples were reheated after the spinning process and isothermally stretched at 150 °C. The corresponding manufacturing parameters and fibre properties are given in Table 1. The spin-draw ratio SDR is given as the ratio of extrusion and winding speed. The (post) draw ratio PDR corresponds to the length relation between the post-drawn and the spin-drawn fibre. Since fibres 1 and 4 in [11] were produced with the same processing conditions and only different stretching, the latter has been renamed into 1* in this study.

### 2.3. Radius Dependence of the Temperature Profiles during Cooling

**Figure 1:**
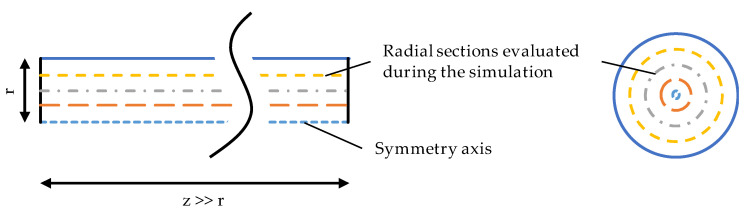
Simplified two-dimensional fibre geometry investigated by numerical simulation (left) and schematic illustration of radial sections evaluated during the simulation.

To investigate the temperature profile within the fibre during the cooling process in the water bath, a numerical simulation was conducted in Comsol Multiphysics, COMSOL Inc., Burlington, VT, USA. The boundary conditions were selected based on the process parameters in Table 1 to approximate the cooling process of the produced fibres. The fibre geometry was assumed to be two-dimensional with a constant spinning radius of $r=d_{0}/2$ and a length of z=50 cm (cf. Figure 1). The short sides of height r, of the resulting rectangle represent the points at which the fibre enters and exits the water bath.

An axis of symmetry is defined over the length of the fibre at r=0 and the edge at $r=d_{0}/2$ represents the outer surface of the fibre surrounded by water. The heat transfer between water and fibre is described by the coefficient $a=Nuk_{w}/d_{0}$, where $Nu=0.76Re^{0.38}$ represents the Nusselt number taken from literature [16] and $k_{w}$ the thermal conductivity of water. The temperature profile inside the fibre is modelled in reference to the equation proposed for melt spinning by Dietz [17], while neglecting crystallisation effects:

(1) $$\frac{dT}{dz}=\frac{1}{v_{z}}\frac{k_{p}}{\rho_{p}c_{p}}\left(\frac{\partial^{2}T}{\partial r^{2}}+\frac{1}{r}\frac{\partial T}{\partial r}\right)$$

Here, $v_{z}$ represents the speed at which the fibre passes through the water bath, whereas $k_{p}$, $\rho_{p}$, and $c_{p}$ represent the thermal conductivity, density, and specific heat capacity of the polymer. The temperature $T_{in}$ at the inlet is set equal to the temperature of the melt. All parameters and their values used in the simulation are given in Table 2.

The steady-state simulation was performed for the samples given in Table 1. As the process parameters for samples 1 and 1* are identical, only one simulation was performed to investigate their cooling process. In the following, the results of the simulation for sample 2 with a quench temperature of $T_{water}=250$ K are given.

**Figure 2:**
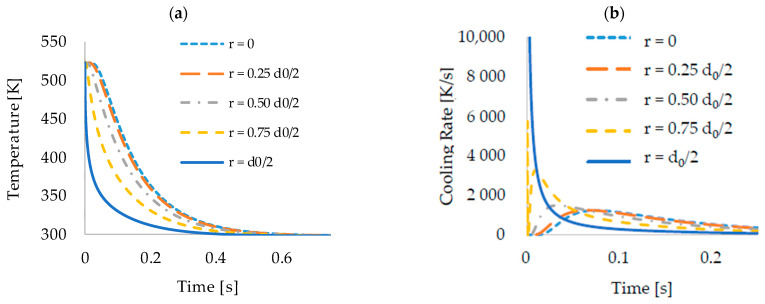
Temperature of the fibre over the time (**a**) it has spent in the bath and the resulting cooling rates with respect to time (**b**). The results show the different rates of cooling that arise inside the fibre depending on the radial location.

As expected, the simulation shows a loss in temperature, increasing rapidly in intensity from the centre of the fibre to its surface, which can be seen in Figure 2a. While the difference in the temperature profile between the centre of the fibre and $r=0.25d_{0}/2$ is small, it is much more significant within $r=0.75d_{0}/2$ and the fibre surface. This can be explained by a higher heat transfer at the surface compared to the heat conduction inside the fibre and agrees with results from previous investigations [15]. After a length of 5 cm, the interior of the fibre has reached about the same temperature as the quench. As for the temperature profiles, the filament velocity was used to determine cooling rates as a function of the change in temperature over time, as seen in Figure 2b. Outliers around t=0 can be attributed to numerical errors in the calculation of the first cells near the inlet. The graphs again highlight the much higher cooling rates near the fibre surface. The cooling rate at the outer surface of the fibre ($r=d_{0}/2$) resembles a decreasing exponential function with an initial value of more than 10^4^ K/s. This quantifies the very rapid cooling caused primarily by the effect of convective heat transfer between polymer melt and water. The other curves show, on the one hand, significantly lower cooling rates and, on the other hand, initially an increase and later a likewise decreasing exponential course of the cooling rate. This can be explained by the comparatively much slower effect of radial heat conduction in PMMA, which explains the effect of the graded refractive index mentioned at the beginning. The data shows that the cooling process at the fibre surface has already progressed considerably before maximum rates are reached inside the centre of the fibre.

## 3. Fibre Analysis via Small-Angle X-ray Scattering Experiments

**Figure 3:**
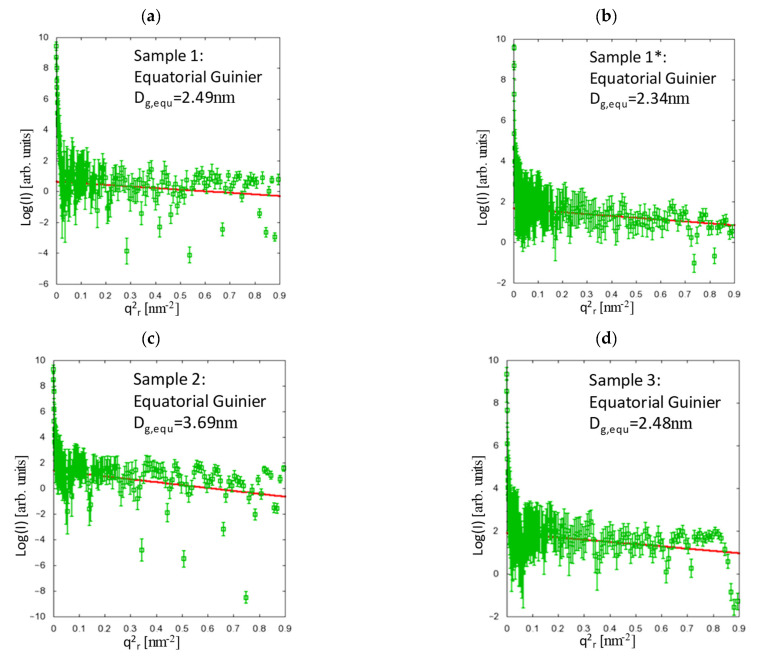
Analysis of the equatorial scattering contributions using the Guinier fit for sample 1 (**a**), sample 1* (**b**), sample 2 (**c**) and sample 3 (**d**). The results show that the equatorial dimensions appear to be dependent on the spin-draw ratio rather than the draw ratio.

The four PMMA-POFs were investigated by small-angle X-ray scattering. The SAXS experiments were performed at the GALAXI-beamline at Forschungszentrum Jülich using a wavelength of 1.34 Å and a sample to detector distance of SDD = 3.6 m. A detailed description of the GALAXI-beamline is provided in [18]. The SAXS patterns were found to be anisotropic with very strong signals only in the meridional and equatorial directions (see Figures 3 and 4 in [11]). Therefore, the analysis of the SAXS intensity distributions was carried out along and perpendicular to the fibre axis. In order to obtain information on the PMMA fibre nanostructure, a Guinier approach as well as the computation of distance distribution functions was applied to the measured SAXS intensities along the fibre axis and the equatorial plane (see Table 2 in [11]). Real-space correlation peaks were obtained in both equatorial and meridional distance distribution functions which suggested periodic variations of the scattering density along and perpendicular to the fibre axis in the amorphous POFs. The resulting structure parameters could be assigned to scattering density variations over the fibre cross-section and the occurrence of straight PMMA chain segments along the fibre axis. The integral structure parameters were found to be clearly correlated to changes in the processing conditions. A model that describes the radially decaying periodic density variation could reproduce the essential structural features of the equatorial scattering curves (see Equations (9)–(11) in [11]), and, therefore, indicated that the origin of the fibre cross-sectional refractive-index profiles is definitely a radial density gradient.

In the current study, the four POF samples were re-examined. In contrast to the preceding investigations, the Guinier dimensions of the four samples in the high-q-regions were analysed, which are important to obtain information on the chain–chain interactions in the fibre (i.e., the entanglement of the polymer chains, and their orientation), and this time the model function defined in [11] was applied to all four fibre samples to examine the drop-off region of the scattering densities. Though the model function is far too simple to fit the SAXS-intensities perfectly, it can be used to estimate the thickness of the region of decaying densities. The results are explained by simulations of the temperature inside the fibres, as well as on the interaction of neighbouring polymer chains in dependence of the temperature. From these investigations, we may yield a clear picture on the interrelationships between the drop-off regions of the densities, the spin-draw ratios, the draw ratios, and the fibre radii.

For the Guinier approximation,

(2) $$I_{\mathrm{obs}}\;\left(q\right)=\;I_{0}\exp\;[-\frac{q^{2}R_{g}^{2}}{3}]$$

where *I_0_* is the forward scattering intensity, *q = 4πsin(θ)/λ* is the modulus of the scattering vector, where *2θ* is the scattering angle, and λ is the wavelength. The Guinier radius *R_g_* is interpreted as a prism with edges A, B, C [12]. In this study, *A = B = D_g,equ_* in the equatorial region, and *C = D_g,mer_* along the fibre axis, i.e.,

(3) $$R_{g}^{2}=\;\frac{D_{g,equ}^{2}}{6}$$

and

(4) $$R_{g}^{2}=\;\frac{D_{g,mer}^{2}}{12}$$

**Figure 4:**
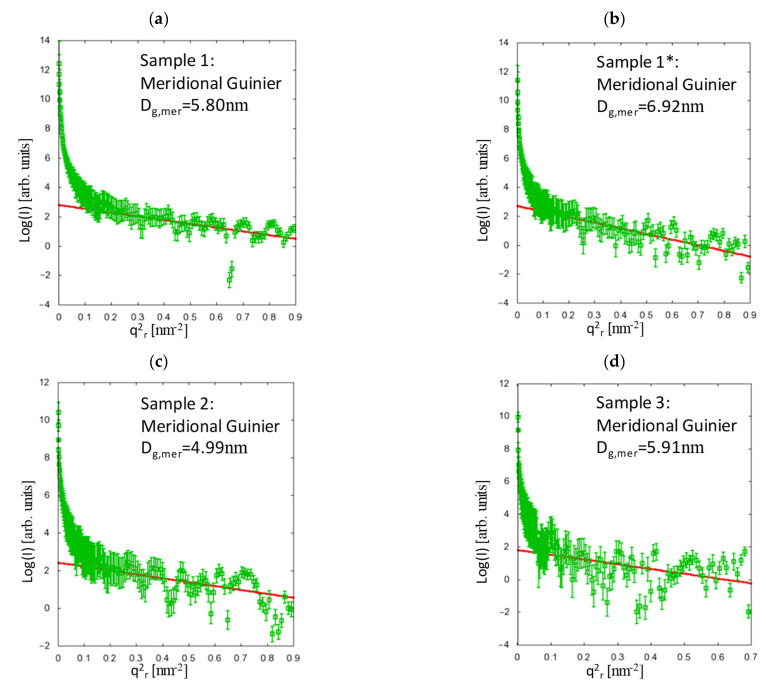
Analysis of the meridional scattering contributions using the Guinier fit for sample 1 (**a**), sample 1* (**b**), sample 2 (**c**) and sample 3 (**d**). The meridional dimensions depend, as expected, on both the spin-draw ratio and the draw ratio.

The results of the Guinier analysis are depicted in Figure 3 and Figure 4, where *q_r_* and *q_z_* are the momentum-transfer vector components in the equatorial plane (fibre cross-section) and along the fibre axis, respectively. There are two samples with smaller fibre diameter (samples 1 and 1*), and two samples with larger fibre diameter (samples 2 and 3, see Table 1), which can be directly compared.

**Figure 5:**
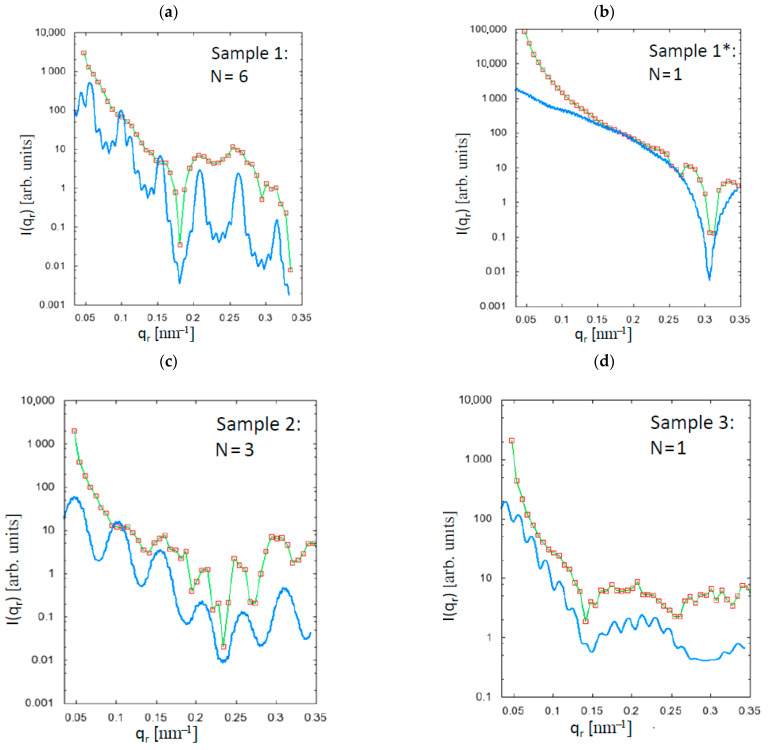
Analysis of the equatorial scattering contributions (red squares and green curves) using the model function (blue curves, see [11]) for sample 1 (**a**), sample 1* (**b**), sample 2 (**c**) and sample 3 (**d**). The parameters Δ1 and Δ2 were manually found by variation of the respective values derived from the equatorial Patterson function within their uncertainties, the values Δρ_1_ and Δρ_2_ are constants as given in [11]. The results show that strong drawing leads to a shrinking of the region ΔR = N(Δ_1_ + Δ_2_) where the densities decay (see Table 3).

As expected, the meridional lengths are increased by both the spin-draw ratio and by the additional drawing, whereas the equatorial dimensions appear to depend only on the spin-draw ratio and not on the draw ratio, which reveals the comparison of samples 1 and 1* in Figure 3 and Figure 4. Here, the equatorial dimensions are larger for smaller spin-draw ratios (see Table 3), since the orientation of the entangled chains becomes different. This may lead to a different density over the fibre radius because the chains experience the tension induced by the spin-draw ratio much stronger at low temperature, i.e., in the solid state, which is at the rim of the fibre, whereas at high temperature, i.e., close to the core, the polymer is yet in a liquid phase. Moreover, any spin-draw induced effects regarding the polymer chain interaction are rather frozen at the rim of the fibre due to the rapid cooling while the polymer chains in the still liquid polymer melt close to the core can relax those effects because after the fibre shell has been cooled below glass transition temperature no spin draw effects can be induced anymore. Considering the results from the model function (see Figure 5 and Table 3), it is clearly shown that the drawing, which is performed above the glass transition temperature, obviously leads to an equalisation of the densities over the fibre radius, which is also reflected in the equatorial dimensions, since after the stronger drawing, the differences in the equatorial dimensions (between samples 3 and 1*) are very small.

For the strongly drawn fibres (samples 1* and 3), the thickness of the region Δ*R* where the densities decay is just the rim of the fibre, whereas for the less-strongly drawn fibres (samples 1 and 2), the region is somewhat extended (see Table 3). The differences in the thickness of the drop-off region between the fibres with smaller (sample 1) and larger (sample 2) radii may be due to the fact that the temperature inside the larger fibre remains higher for a longer time, which may also lead to an equalisation of the densities, i.e., the orientations of the polymer chains become more and more similar over the fibre radius, a fact that is also observed in the polymer simulations in Section 4. The period of higher temperature inside the fibre, however, also depends on the winding speed, i.e., on the spin-draw ratio, as well as on the water bath temperature *T_water_.*

The results indicate that for a gradient index POF, an appropriate fibre radius (spin-nozzle diameter, so that the temperature inside the fibre can be better regulated), spin-draw ratio, and water bath temperature (for the temperature control inside the fibre) is required. Additional strong drawing of the fibre above the glass transition temperature should not be performed in order to avoid the equalisation of the densities over the fibre radius. The fact that all of the investigated fibres have been drawn, may serve as an explanation for the very small Δ*R*-values, i.e., the fibres are either multistep index (MSI, sample 1 and 2) or single step index (SI, sample 1* and 3) POFs. By modifying the temperature over the fibre radius (via the spin-draw ratio), it appears to be possible to increase or decrease the region where the densities drop-off.

## 4. Numerical Analysis of the Structural Properties

In order to better understand the cooling process and the formation of the final polymer structure, the polymer was modelled using a Monte-Carlo simulation approach [19]. The 15 calculated temperature profiles (five profiles per three different fibres) from Section 2 were used as input for the simulation of the structural properties.

### 4.1. Monte-Carlo Simulation

**Figure 6:**
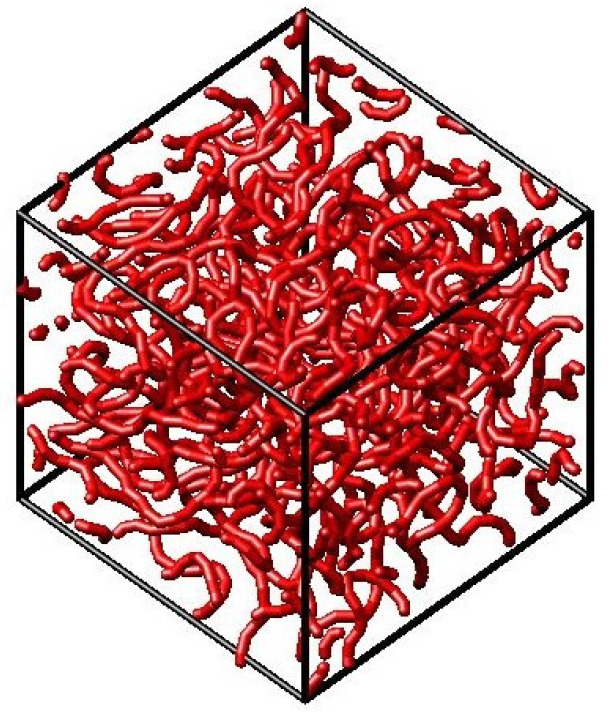
Illustration of an exemplary three-dimensional simulation volume with 200 polymer chains consisting of 200 monomers each.

The Monte-Carlo (MC) simulation is a statistical method. The three-dimensional simulation volume was, therefore, discretised into 50 × 50 × 50 positions and the polymers were assumed to be chains of monomers, which occupy discrete and neighbouring places within this volume. An illustrative example is shown in Figure 6. A periodic boundary condition was applied in order to emulate a homogenous and infinitely large volume. A coarse-graining model has been developed to simplify the polymer complex structure [20]. Coarse-graining (CG) technique is known to reduce computation time by removing the intra-monomer details, which is done by groping some atoms of the monomer to a single bead. Thus, 200 polymer chains were assumed with 200 beads each.

First, an initial constellation was generated by random placing of the polymers and their beads within the simulation volume. After the first bead was placed randomly into the empty space, each new bead was grown using a configurational bias, which means that many orientations were analysed and the one of highest probability was chosen. During this process, the initial temperature was considered. The main advantage of such method is it generates a polymer chain with a minimal energy close to the relaxed-chain energy, which minimises the number of steps to reach the relaxation of the chain. After the full configuration was generated, the constellation was simulated assuming a pressure of 1 bar and a constant temperature, which was the starting point of the temperature profile to be considered. This leads to a further relaxation.

The Monte-Carlo simulation goes in cycles. In each cycle, all polymer chains are treated, one after another. For each chain, the monomers were serially chosen and evaluated, which movements to unoccupied neighbouring places were possible without polymer crossing. For these potential new positions *n*, the resulting energy *E_n_* was calculated and the probability that this energy state can be taken. Higher energies were less likely to be occupied than lower ones, but the probabilities depend on the actual temperature:

(5) $$p=\min\left(1,\exp\left[-E_{n}-\frac{E_{0}}{k_{B}T}\right]\right),$$

where *E_n_* is the energy at displaced location and *E_o_* is the energy in the old position with the Boltzmann constant *k_B_* and temperature *T*. A higher temperature favours higher energy levels, and vice versa. For each monomer, a random potential movement was assumed, a random number between 0 and 1 was chosen, and the movement was accepted if the number was lower or equal to the calculated probability. Otherwise, the movement was declined and the monomer remained on the original spot. This considers that higher energy levels can be taken, but with an ever-lower probability. Here, the temperature varies over time according to the temperature profile, which is taken from the thermodynamic simulations of Section 2. Using the obtained temperatures at the respective time instances instead of cooling ratios prevents the evaluation of numerical derivatives and increased the accuracy. The time steps were adaptively chosen according to the change to the constellation. Therefore, the number of realised accepted movements according to Equation (5) was taken and compared to the number of total attempts. If the acceptance ratio exceeded 50% the time step was decreased, otherwise it became slightly longer. The simulation was performed until the end of the temperature profile was reached resulting in more than 10,000 Monte-Carlo cycles on average. Each simulation was repeated six times and mean and standard deviation were evaluated.

In order to represent PMMA as polymer, material parameters and potentials for the energy calculation were used according to [20,21]. The most important parameters are listed in Table 4.

The simulations were performed for 15 temperature profiles, which resulted in molecule configurations that represent the local distribution of the polymer chains within the volume. Starting from these microscopic distributions, macroscopic effects such as the density and the cross-chain interaction were evaluated.

### 4.2. Density Variation

The polymer density ρ is simply the mass of all monomers in a unit volume. In order to consider the special behaviour of polymer chains, however, the method of the radius of gyration was used [22]. Here, the space/volume, which is occupied by each polymer chain, is counted as a kind of orbital. Since each polymer is modelled by number of connected beads, it can be said that effective occupied space is represented by a sphere in 3D or circle in 2D that enclosed the polymer chain. The density of each polymer in 3D is calculated for each position by the number of spheres *N* to which this point belongs to:

(6) $$\rho =\frac{3\cdot m_{mono}\cdot N}{4\pi R_{G}^{3}},$$

where *R_G_* is the radius of the sphere and *m_mono_* the mass of a monomer. However, once two polymer chains are in approximate distance of each other, both chains will be hindered.

**Figure 7:**
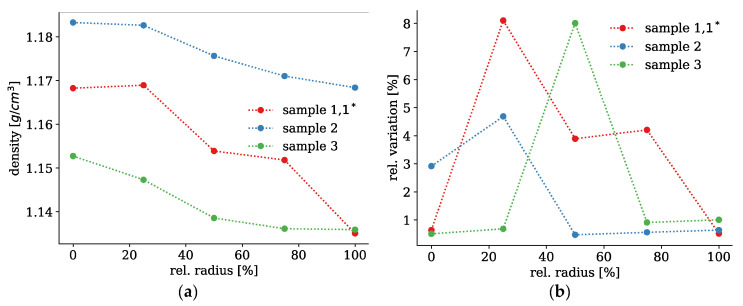
Simulated density profiles over the radius of the investigated samples (**a**). The densities show a clear decrease towards the outer regions. The relative density variation follows no real trend, but sample 2 shows the most homogeneous density (**b**).

The evolution of the simulated densities over the radius is plotted in Figure 7 on the left. All densities decrease from the centre of the fibre towards the surface. Sample 2 shows the highest density overall, whereas samples 1 and 1* with the smallest spinning diameter feature the most pronounced decrease of the density. Sample 2 features the largest spinning diameter (0.47 mm) and cools down more slowly. However, it is also cooled for the longest time due to its slow spinning speed. Samples 1 and 1*, however, are the thinnest and drawn at the highest speed. Thus, there is only a short cooling time, but sufficient to generate a profile in such a thin filament. Sample 3 has an intermediate spinning diameter of 0.28 mm, but is treated at warmer temperature for a longer time, which resulted in a relatively narrow profile in the inner part around the axis. Only here, the cooling time was sufficient for a better alignment of the polymers resulting in a slight increase of density. The density variation indicates potential inhomogeneities that give rise to scattering. None of the three samples showed a particular trend, but a slight increase of variation in the region where the density profiles started.

In summary, all samples feature density profiles that decrease towards the surface, but they are different in height and width. Samples 1, 1* show the broadest, but shallowest, and sample 3 the narrowest profile in relation to the filament diameter, which are in line with the SAXS measurements. Considering the large diameter of 0.47 mm, sample 2 features a more gradual decrease than samples 1, 1*. Sample 3 with the same filament diameter is also steeper and narrower resembling more a step-index (*N* = 1). The variations increase in those regions where the density profiles either start or end, where structural transitions occur.

### 4.3. Chain Interaction

With increasing density, the polymer chains lay closer to each other and will show a stronger interaction. This influences mechanical properties such as e.g., stiffness, but also optical effects. Influenced by the SAXS results, we analysed how neighbouring chains interact to each other. The SAXS measurements indicate that there is a transition region towards the fibre centre from which on the polymers were much more aligned giving rise to elevated scattering.

**Figure 8:**
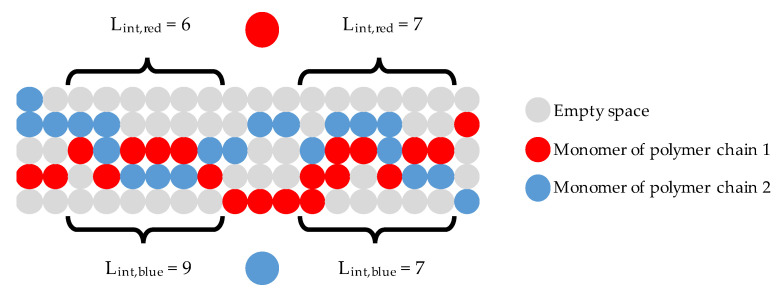
Two-dimensional illustration of the interaction length *L_int_*.

In the numerical evaluation, this alignment was quantified by an interaction length L_int_ being the total number of directly adjacent monomers of two particular neighbouring polymers that hinder each other’s movement. Thus, the interaction length is always evaluated in pairs of two polymer chains influencing each other by occupying a certain number of adjacent positions and thus reducing the number of possible movements. Figure 8 illustrates the idea of the interaction length in two dimensions.

In order to quantify the total amount of cross-chain interaction within the material, all interaction lengths between each combination of two polymer chains were statistically analysed and histograms of all occurring interaction lengths were produced.

**Figure 9:**
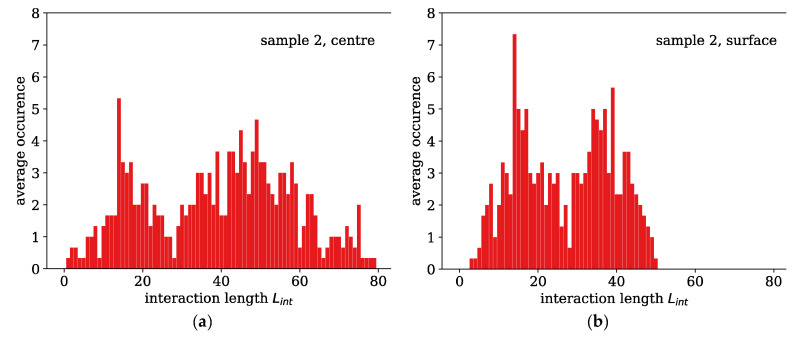
Two exemplary histograms of the interaction lengths in sample 2 at the fibre axis (**a**) and the surface (**b**). There is an obviously larger alignment near the fibre axis.

Figure 9 shows examples for two histograms of sample 2 at the centre and the surface of the fibre. There are two obvious observations to be made: the histograms feature a particular shape with two distinct maxima. This is a special property of PMMA and seems to come from the bending potential, which favours two distinct angles. Secondly, the histograms are obviously broader in the inner region with more interactions and alignment between the chains.

**Figure 10:**
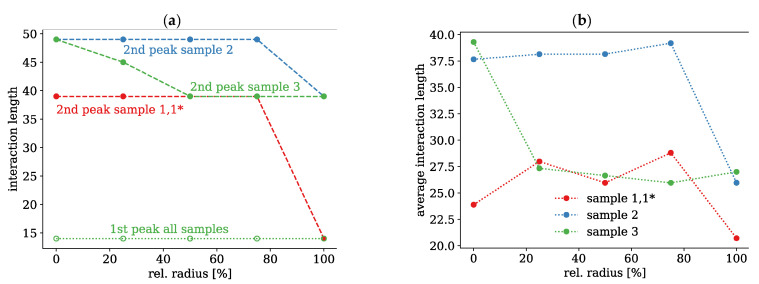
Location of the two maxima in the histograms (**a**) and simulated evolution of average interaction lengths over the radius of the investigated fibre samples (**b**).

Due to these observations, all histograms of the interaction lengths were analysed with respect to the location of the two maxima and the average interaction lengths. All histograms of the interaction lengths show two maxima, which were examined more closely. The locations of these maxima are plotted in Figure 10a for all three samples and radius positions. The first maximum at shorter interaction lengths is constant for all samples and does also not change over the radius. This seems to be a property of PMMA and independent of the cooling profile. The second peak at longer interaction lengths, however, differs and is strongly influenced by the temperature profile of the cooling process. At the inner regions of all three samples, this second peak occurs at longer interaction length, whereas the two peaks become closer for faster cooling at the surface. The largest difference can be observed for thickest sample 2, the smallest for the thinnest sample 1,1*.

The average interaction lengths for the three investigated fibres are shown in Figure 10 on the right. There is a slight trend towards stronger interactions near the centre of the fibre. Sample 2 with the highest density also exhibits the strongest interactions. This is to be expected, since the polymer chains are packed more closely. However, there is no strict relation between density and chain interaction. Sample 3 for instance, shows a very strong interaction at the centre, which is larger than for sample 2, whereas the density is lower at this point. Here, the three samples show a quite different behaviour, which is consistent with the simulated densities, but also with the SAXS measurements. It appears that there are two regions of lower and higher chain interaction. For sample 2, the transition between these two regimes lies at about 75% of the radius or about 120 µm from the surface. In sample 3, the transition is much closer to the centre leading to a narrower region, but has more or less the same distance to the surface due to the smaller diameter. In sample 1, 1*, however, the chain interaction remains relatively low over the entire radius, which may be attributed to the small radius and the fast cooling throughout the entire fibre cross section.

## 5. Conclusions

**Figure 11:**
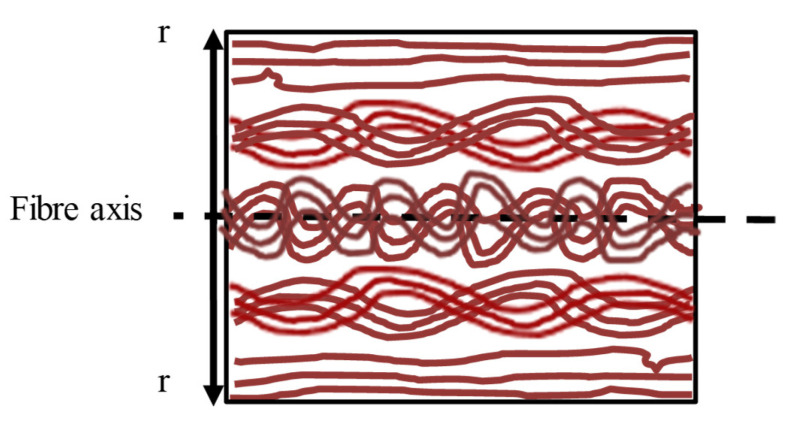
Schematic illustration of radius-dependent polymer chain interaction (and orientation; differences exaggerated for clarity).

Four PMMA-POFs produced with different sets of process parameters were re-investigated with small-angle X-ray scattering and compared to polymer Monte-Carlo simulations of the cooling. The SAXS intensity distributions were analysed by a Guinier approach in the high-q regions and the distance-distribution functions were computed to extract information on the PMMA fibre nanostructure in the equatorial plane and along the fibre axis. Regions of higher density and polymer–chain interaction could be found in the inner regions of the fibres. Post drawing above glass-transition temperature led to an equalisation of the density profile, whereas the choice of the spinning diameter, winding speed and water temperature had a profound impact on the molecular structure. A larger spin-draw ratio and larger differences of the cooling speeds within the filament resulted in an elevated entanglement in the inner region of the fibre and indicated to different regimes in the inner and outer part of the filament. Numerical investigations by Monte-Carlo simulations confirm the density profile over the fibre radius. Moreover, they also indicate a region of higher chain interaction in the inner region of the fibre with similar transition radii to observations in the SAXS measurements. Measurement and simulation indicate a polymer chain structure within the fibre, as schematically shown in Figure 11. Radial differences in the interaction length of the polymer chains and in the density are both induced by the effect of rapid cooling in the water bath. The numerical simulations indicate, however, that the effects are not directly related to each other and can occur to different degrees depending on the process.

The statistical evaluation of the interaction lengths for different cooling profiles within the investigated samples at different radius positions also revealed two rather discrete interaction lengths with high occurrence. The shorter seems to be independent of the cooling conditions and rather a material property, whereas the maximum at higher interaction lengths is clearly process dependent.

## Figures and Tables

**Table 1:** Fibre processing conditions of the four polymer optical fibre (POF) samples (see [11] for explanations).

No.	Nozzle Diameter *d_N_* (mm)	Extrusion Speed *v*_E_ (m/min)	Spin Draw Ratio SDR	Winding Speed *v*_W_ (m/min)	Post Draw Ratio PDR	Quench Temperature T_water_ (°C)	Spinning Diameter *d_0_* (mm)	Fibre Diameter d_Final_ (mm)
1	0.8	2.39	18.85	45	3	25	0.18	0.06
1*	0.8	2.39	18.85	45	7	25	0.18	0.3
2	2.0	0.38	18.32	7	3	25	0.47	0.16
3	2.0	0.38	52.36	20	5	35	0.28	0.06

**Table 2:** Parameters used for the numerical simulation of the temperature profiles during cooling.

Parameter		Value	Unit
Density of the polymer	$\rho_{p}$	1180	kg/m^3^
Specific heat capacity of the polymer	$c_{p}$	1450	J/(kg K)
Thermal conductivity of the polymer	$k_{p}$	0.19	W/(m K)
Density of water	$\rho_{w}$	1000	kg/m^3^
Specific heat capacity of water	$c_{w}$	4183	J/(kg K)
Thermal conductivity of water	$k_{w}$	0.5984	W/(m K)
Viscosity of the water	η	1	mPa s
Temperature at the inlet	$T_{in}$	523.15	K

**Table 3:** Manually obtained equatorial dimensions Δ_1_ and Δ_2_ of the investigated POF samples, number of repeat units N, and thickness of the region of decaying densities ΔR.

Sample No.	Δ_1_, (nm)	Δ_2_, (nm)	N	ΔR = N (Δ_1_ + Δ_2_) (nm)
1	85 (3)	35 (3)	6	720 (24)
1*	81 (3)	21 (3)	1	102 (4)
2	94 (3)	27 (3)	3	363 (18)
3	66 (3)	43 (3)	1	109 (4)

**Table 4:** Overview of the most important Monte-Carlo simulation parameters [16,17].

Parameter	Value
Bond potential K_bo_	184.47 kcal/mol K
Bond length L_0_	2.659 Å
Bend potential K_be_	80.6 kcal/mol rad
Bend potential $\vartheta_{0}$	2.771 rad
Torsion constant K_torr,0_	0
Torsion constant K_torr,1_	0.636 kcal/mol rad
Torsion constant K_torr,2_	−129 kcal/mol rad
Torsion constant K_torr,3_	1.5 kcal/mol rad
Lennard–Jones potential ε	0.277676 kcal/mol
Lennard–Jones potential ρ	3.632093 Å

## Data Availability

The data presented in this study are available on request from the corresponding author.
